# Eine Bestandsaufnahme zur Klimakrise inmitten der COVID-19-Pandemie

**DOI:** 10.1007/s00502-020-00833-6

**Published:** 2020-10-15

**Authors:** Harald E. Rieder

**Affiliations:** Institut für Meteorologie und Klimatologie, Universität für Bodenkultur, Gregor-Mendel-Straße 33, 1180 Wien, Österreich

**Keywords:** Klimawandel, Treibhausgase, COP21, Anthropozän, climate change, greenhouse gases, COP21, Anthropocene

## Abstract

Die atmosphärische Konzentration gutgemischter Treibhausgase ist seit 1850 drastisch angestiegen. Hauptursache hierfür ist menschliches Handeln, vor allem die Verbrennung fossiler Energieträger. Die Auswirkung dieser Veränderung der chemischen Zusammensetzung der Erdatmosphäre ist ein positiver Strahlungsantrieb, welcher sich letztendlich in der beobachteten Erderwärmung manifestiert. Die globale Mitteltemperatur hat seit der vorindustriellen Zeit um ca. 1,0 °C zugenommen und bei fortschreitender Emission von Treibhausgasen auf heutigem Niveau droht eine Temperaturzunahme bis 2100 von 3,7 °C bis 4,8 °C über dem Durchschnitt von 1850–1900. Ein derartiger Temperaturanstieg wäre mit fatalen Folgen für viele Ökosysteme und auch uns Menschen verbunden, wie die Sachstandsberichte des Weltklimarats dies eindrücklich darlegen. Aus diesem Grund wurde auf der Pariser-Klimakonferenz beschlossen, die globale Erwärmung auf unter 2 °C, idealerweise auf 1,5 °C gegenüber dem vorindustriellen Niveau zu begrenzen. Um dieses Ziel zu erreichen, sind rasche und drastische Emissionsreduktionen hin zu Netto-Null-Emission spätestens bis 2050 erforderlich. Ein Blick auf die Entwicklung der globalen Treibhausgasemissionen der letzten Jahre und Jahrzehnte zeigt deutlich, wie weit wir von diesem Ziel entfernt sind, steigen die globalen Emissionen doch immer noch an. Auch die im Rahmen der Pariser Klimaziele zugesagten nationalen Emissionsreduktionen sind auf momentanem Stand nicht ausreichend, um das 1,5-°C-Ziel zu erreichen. Es ist nun höchste Zeit, die Anstrengungen deutlich zu erhöhen. Noch ist die Erreichung des 1,5-°C-Ziels möglich, die Zeit, um die erforderlichen Maßnahmen zur Emissionsminderung zu setzen, läuft aber binnen dieses Jahrzehnts ab.

## Abstract (en)

The atmospheric concentration of well-mixed greenhouse gases has drastically increased since 1850. The prime cause for this increase is anthropogenic activity, particularly the burning of fossil fuels. As a consequence of the changing atmospheric composition, we observe a net positive radiative forcing, which manifests in global warming. The global mean surface temperature has increased since the preindustrial by about 1.0 °C. Under the assumption of continued greenhouse gas emissions, climate models project a temperature increase between 3.7 °C and 4.8 °C until 2100 (compared to the 1850-1900 mean). The assessment reports of the Intergovernmental Panel on Climate Change detail the catastrophic consequences of global warming of such extent for both ecosystems and mankind. As a consequence, the Paris Agreement aims to limit global warming to below 2 °C, ideally 1.5 °C, when compared to the preindustrial. To achieve this goal, fast and ambitious emission controls are required, reaching net zero emission by 2050 at the latest. Examining the global greenhouse gas emissions of recent decades, it becomes obvious how far away we are at present from reaching this goal. Also, the currently determined national contributions for emission reduction do not suffice to meet the 1.5 °C target. Thus, it is of uttermost importance to raise the global ambition in climate protection. The 1.5 °C target can still be reached, however, the time to set the required measures will expire within this decade.

## Einleitung

Vor nunmehr fast zwei Jahrzehnten erschien ein vielbeachteter Fachaufsatz des Nobelpreisträgers Paul Crutzen, in welchem dieser vorschlägt, die Epoche beginnend mit dem späten 18. Jahrhundert als Anthropozän zu bezeichnen [1]. Crutzen wählt diesen Zeitpunkt, da Isotopenanalysen von Luft, die im polaren Eisschild eingeschlossen ist, mit Ende des 18. Jahrhunderts deutliche Zuwächse in der atmosphärischen Konzentration von Kohlendioxid (CO_2_) und Methan (CH_4_) aufzeigen. Er beschreibt weiter, wie sich das Bevölkerungswachstum und der Energie- und Ressourcenverbrauch über die folgenden Jahrhunderte beschleunigte, die anthropogenen Emissionen sich vervielfachten, sensible Ökosysteme zerstört wurden, und das Artensterben zugenommen hat. Crutzen betont auch drastisch die Magnitude und Persistenz des eingeleiteten Klimawandels und das Ausmaß, in welchem die Menschheit in die Umwelt eingreift, und unter der Annahme des Ausbleibens einer globalen Katastrophe (z.B. Meteoriteneinschlag, Weltkrieg, Pandemie), über die nächsten Jahrhunderte und Jahrtausende wird.

Crutzens Zusammenfassung aus dem Jahr 2002 basiert auf den Erkenntnissen des dritten Sachstandsberichts des Weltklimarates (Intergovernmental Panel on Climate Change, IPCC); die Kernaussage seiner Synthese hat aber heute durch Fortschreiten der globalen Erwärmung und der damit verbundenen Umweltveränderungen unverändert Gültigkeit. Wie sehr der Mensch seit der Industrialisierung in das Klimasystem eingegriffen hat, wird umfassend in den Sachstandsberichten des IPCC dokumentiert, deren Sprache über die Jahre stark an Deutlichkeit zugelegt hat, wie nachstehend exemplarisch anhand von Aussagen aus den jeweiligen Zusammenfassungen für politische Entscheidungsträger ausgeführt ist.

Hieß es im 2001 erschienen 3. Sachstandsbericht noch „Die Abwägung der Erkenntnisse legt einen erkennbaren menschlichen Einfluss auf das globale Klima nahe“ [2], so benennt der 4. Sachstandsbericht die Rolle des Menschen an der Erderwärmung bereits eindeutig: „Der größte Teil des beobachteten Anstiegs der mittleren globalen Temperatur seit Mitte des 20. Jahrhunderts ist sehr wahrscheinlich durch den beobachteten Anstieg der anthropogenen Treibhausgaskonzentrationen verursacht“ [3]. Noch umfassender wird dies im vor einigen Jahren erschienen 5. Sachstandsbericht formuliert: „Der Einfluss des Menschen auf das Klimasystem ist klar. Das ist offensichtlich aufgrund der ansteigenden Treibhausgaskonzentrationen in der Atmosphäre, dem positiven Strahlungsantrieb, der beobachteten Erwärmung und des Verständnisses des Klimasystems“ [4].

**Abb. 1.**
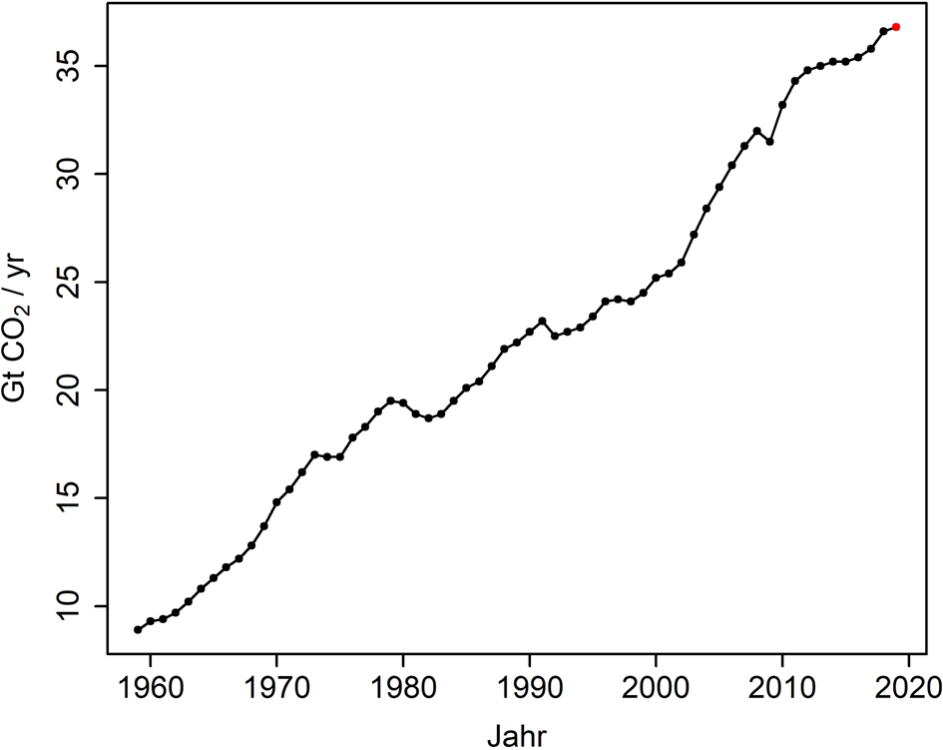
Entwicklung der globalen fossilen CO_2_-Emissionen von 1959-2018 inklusive der Schätzung für 2019. Darstellung basierend auf Daten von [7, 8]

Die globale Erwärmung als physikalische Folge des durch menschliches Handeln stetig steigenden Treibhausgaskonzentrationen veränderten Strahlungshaushalts unseres Planeten ist heute in wissenschaftlichen Kreisen unbestritten [5]. Ebenso, dass nur eine deutliche Reduktion der Emissionen und letztlich Stabilisierung der Treibhausgaskonzentrationen das Ausmaß der Klimakrise begrenzen kann [6]. Trotz vielfältiger Beschlüsse und Abkommen sind die Treibhausgasemissionen in den letzten Jahren jedoch global deutlich gestiegen (siehe Abb. 1). Ein anschauliches Beispiel: im Jahr 2019 übertrafen die CO_2_-Emissionen jene des Jahres 2015 (UN-Klimakonferenz (COP21^1^) in Paris) um 4 %, und jene des Jahres 1990 (Veröffentlichung des 1. IPCC Sachstandsberichts) um mehr als 60 % [7].

Aus der Analyse der Zusammensetzung des in Eisbohrkernen eingeschlossenen Luftgemischs wissen wir, dass Treihausgaskonzentrationen, wie wir sie heute in der Atmosphäre vorfinden, zumindest in den letzten 800.000 Jahren nicht aufgetreten sind und dass der Anstieg der CO_2_-Konzentration in den letzten Jahrzehnten um ein vielfaches schneller von statten ging als in früheren Epochen der Erdgeschichte [9]. Die Erderhitzung, wie wir sie beobachten, ist weitestgehend eine direkte Folge dieses Eingriffs von uns Menschen in das Klimasystem. Die Veränderung des globalen Strahlungshaushalts und folglich der Mitteltemperatur ist aber nur eine von vielen Veränderungen, die über die letzten Jahrzehnte aufgetreten sind. Abschnitt 2 bietet einen Auszug der vielfältigen Konsequenzen unseres Handels für Elemente des Klimasystems.

## Veränderungen im Klimasystem und Klimafolgen

Die globale Mitteltemperatur dient weithin als praktische Maßzahl für den Grad der Erderhitzung, hat sie doch seit der vorindustriellen Zeit um ca. 1,0 °C zugenommen. Die Änderungen im Klimasystem durch Eingriffe des Menschen gehen aber weit über eine Zunahme der globalen Mitteltemperatur hinaus. Beinahe alle Bereiche des Klimasystems sind durch menschliches Handeln beeinflusst. Zu den prominentesten Auswirkungen der Erderwärmung zählen der Rückgang der Schnee- und Eismengen, die Erwärmung und zunehmende Versauerung der Ozeane, und der Anstieg des Meeresspiegels. Besondere Sorge bereiten der massive Masseschwund der Eisschilde in Grönland, die Abnahme der Ausdehnung des arktischen Meereises sowie die fortschreitende Gletscherschmelze [4]. Letztere lässt sich auch deutlich vor unserer Haustür in den Alpen beobachten [10, 11].

Besonderes Augenmerk ist aber nicht nur auf die Magnitude, sondern auch auf die Geschwindigkeit des Klimawandels zu legen, sind doch viele der seit den 1950er Jahren beobachteten Veränderungen davor über Jahrzehnte bis Jahrtausende nie aufgetreten [4]. Auch die globale Gleichzeitigkeit und Geschwindigkeit der Erderwärmung der letzten Jahrzehnte ist einzigartig, zumindest in den letzten 2000 Jahren [12]. Untersuchungen der fünf vorindustriellen Klimaepochen zeigen, dass sich in keiner von diesen ein so kohärentes Bild der globalen Temperaturänderung ergibt wie heute. Auch in der wissenschaftlichen Literatur oft beschriebene spezielle Klimaphasen wie die „Kleine Eiszeit“ (ca. 1300-1850 AD) oder „mittelalterliche Warmzeit“ (ca. 950-1250 AD) erscheinen eindeutig als regionale und nicht globale Phänomene [12]. Diese Analysen zeigen ebenso wie die Ergebnisse der koordinierten Klimamodellierung [4], dass die Rate und globale Verbreitung der Erderhitzung nicht mit natürlichen Schwankungen des Klimasystems erklärt werden kann. Sie ist weitgehend auf die Aktivität von uns Menschen zurückzuführen.

Klimaskeptiker versuchen Zweifel an der Erderhitzung und/oder des Beitrags des Menschen am Klimawandel zu streuen [13, 14]. Hierzu finden verschiedene Taktiken Anwendung, weit verbreitet sind z.B. das Herausgreifen einzelner meteorologischer Ereignisse, Jahre oder Jahreszeiten, oder die Überbetonung der Bedeutung der natürlichen Schwankungen einer Klimagröße (z.B. Sonnenaktivität). Aber einzelne kühle Sommer oder kalte Winter sagen den Klimawandel nicht ab. Sie sind Teil der natürlichen Klimavariabilität.

Hierzu ein aktuelles Beispiel aus Österreich: auch das durchaus örtlich wechselhafte Wetter, welches wir im Sommer 2020 erlebt haben, ist keine Besonderheit in Betracht der natürlichen Klimavariabilität. Das Auftreten einzelner kühlerer oder regnerischer Episoden ist immer möglich, dafür sorgt alleine die Variabilität in den Strömungslagen oder großskaligen Klimamoden. Entscheidend ist die Häufigkeit des Auftretens von Ereignissen bzw. die Änderung deren Auftrittswahrscheinlichkeit. Hier sehen wir eindeutige Tendenzen. Wenngleich der heurige Sommer nicht durch langanhaltende Hitzewellen geprägt war, war er wärmer als 90 % der Sommer in der über 250-jährigen Messgeschichte der Zentralanstalt für Meteorologie und Geodynamik (ZAMG). Entscheidend ist die gesamthafte Betrachtung der Klimaentwicklung der letzten Jahrzehnte, und hier zeigt sich ein eindeutiges Bild: die Mittel-, Maximal- und Minimal-Temperaturen steigen, die Extremereignisse häufen sich, und Veränderungen in Dauer und Beginn der Vegetationsperiode sind zu verzeichnen [15].

**Abb. 2.**
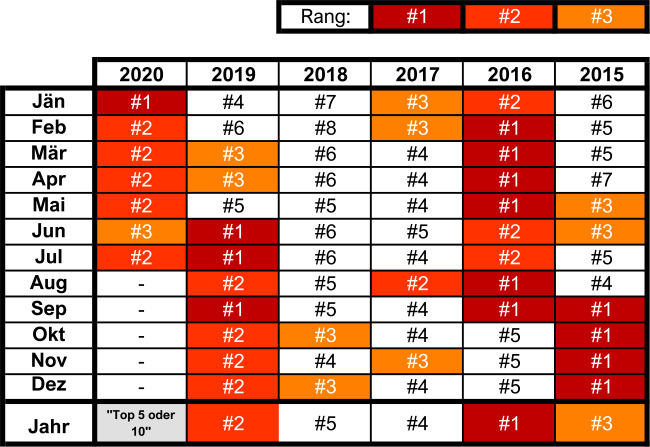
Rangplätze der global gemittelten Jahres- und Monatsmitteltemperaturanomalie (Land+Ozean, in Bezug zum Mittel 1901-2000) für 2015-2020 basierend auf Daten für 1880-2020. Darstellung basierend auf Daten von [17]

Global betrachtet war jedes der letzten drei Jahrzehnte sukzessive wärmer als alle vorangehenden Jahrzehnte seit 1850 [4]. Die 20 wärmsten Jahre sind allesamt seit 1998 aufgetreten, und es zeigt sich flächig eine Zunahme warmer Tage und Nächte, der eine Abnahme kalter Tage und Nächte gegenübersteht. Im Ranking der globalen Jahresmitteltemperaturen rangieren die letzten 5 Jahre allesamt in den Top 5, und auch auf Basis der Monatsmittel lässt sich eine eindeutige Häufung der Extreme in den letzten Jahren feststellen (siehe Abb. 2). Auch der bisherige Verlauf des Jahres 2020 entspricht diesem Bild, und statistische Schätzungen zeigen, dass sich auch 2020 mit sehr hoher Wahrscheinlichkeit in die Top 5 oder zumindest Top 10 wärmsten Jahre einreihen wird [16].

Der Temperaturanstieg ist sicherlich die prominenteste Manifestation des Klimawandels, die Folgen des Klimawandels reichen jedoch weit über die reinen Veränderungen in meteorologischen Größen wie Temperatur und Niederschlag hinaus. Angetrieben von diesen verändert sich unter anderem die Ausbreitung von Vegetationszonen, das Aufkommen und die Verbreitung von Schädlingen sowie die Zusammensetzung und Vielfalt von Arten. Besonders bedeutsam ist hier wiederum die Geschwindigkeit, mit der sich der Klimawandel vollzieht, da vielfach die Veränderung schneller geschieht, als Arten sich an neue Bedingungen anpassen können. Durch die Trägheit des Klimasystems werden auch viele Folgen des Klimawandels für Jahrhunderte andauern, und somit Anpassungsstrategien notwendig, selbst wenn es gelänge die anthropogenen Emissionen heute zu stoppen.

Der 5. Sachstandsbericht des IPCC fasst dies eindrücklich in seinen Kernaussagen zusammen. Diese betonen die Bedeutung von Mitigation und die Existenz von Anpassungsgrenzen. So heißt es im Bericht wörtlich „ohne zusätzliche Minderungsbemühungen, die über heute bestehende hinausgehen, und trotz Anpassung wird die Erwärmung zum Ende des 21. Jahrhunderts zu einem hohen bis sehr hohen Risiko schwerwiegender, weitverbreiteter und irreversibler globaler Folgen führen“ [4]. Bezüglich der Limits der Adaption stellt der Bericht fest: „Anpassung kann die Risiken von Folgen des Klimawandels verringern, allerdings ist ihre Wirksamkeit begrenzt, insbesondere bei größerem Ausmaß und höherer Geschwindigkeit des Klimawandels“ [4]. In diesem Kontext betont der 5. Sachstandsbericht auch die Bedeutung einer längerfristigen Perspektive im Kontext nachhaltiger Entwicklung, da „zeitnahe Anpassungsmaßnahmen auch zukünftige Handlungsoptionen und Vorsorge verbessern“ [4].

An dieser Stelle ist es auch wichtig zu erwähnen, dass die durch die bereits aufgetretenen anthropogenen Emissionen ausgelöste Erwärmung für Jahrhunderte bis Jahrtausende bestehen bleiben wird, jedoch unwahrscheinlich die 1,5 °C Marke überschreiten wird. Im Gegensatz dazu zeigen Kimamodellprojektionen die von keiner zusätzlichen Emissionsminderung ausgehenden Anstiege in der mittleren globalen Oberflächentemperatur im Jahr 2100 von 3,7 °C bis 4,8 °C über dem Durchschnitt von 1850–1900 [4]. Würde eine derartige Erwärmung auftreten, würde dies auch viele sensible Kippelemente des Klimasystems dauerhaft destabilisieren sowie selbstverstärkende Rückkopplungen und Kaskadeneffekte hervorrufen [18]. Im Gegensatz dazu zeigen Modellprojektionen, welche Emissionsszenarien folgen, die zu CO_2_-Äquivalent-Konzentrationen im Jahr 2100 von etwa 450 ppm oder weniger führen, eine wahrscheinliche Erwärmung über das 21. Jahrhundert von weniger als 2 °C gegenüber dem vorindustriellen Niveau [4]. Eine ebensolche Begrenzung der Erderwärmung auf deutlich unter 2 °C, idealerweise auf 1,5 °C, ist das Ziel, dass im Rahmen der Pariser-Klimakonferenz (COP21) am 12. Dezember 2015 ausgerufen wurde.

## Die Bedeutung der Erreichung der Pariser Klimaziele und Wege dahin

Der COP21 folgend nahm der Weltklimarat die Einladung zur Verfassung des Sonderberichts über die Folgen einer globalen Erwärmung um 1,5 °C gegenüber vorindustriellem Niveau und der damit verbundenen globalen Treibhausgasemissionspfade an. Dieser Sonderbericht legt die Auswirkungen globaler Erwärmung um 1,5 °C sowie einen Vergleich zwischen globaler Erwärmung um 1,5 °C und 2 °C dar. Er zeigt eindrücklich, dass bei aktueller Geschwindigkeit die globale Erwärmung 1,5 °C wahrscheinlich zwischen 2030 und 2052 erreicht, und dass die klimabedingten Risiken für natürliche und menschliche Systeme bei einer globalen Erwärmung um 1,5 °C höher als heute, aber geringer als bei 2 °C sind [6]. Modellprojektionen folgend treten in vielen Regionen signifikante Änderungen in Hitzeextremen, Starkniederschlägen bzw. in der Wahrscheinlichkeit für Dürre und Niederschlagsdefizite auf. Auch zeigen Modellprojektionen geringere Folgen für Biodiversität und Ökosysteme bei einer Erwärmung von 1,5 °C als bei 2 °C. Der Sonderbericht zeigt weiters wichtige Synergien zwischen Klimaschutz und den Zielen für nachhaltige Entwicklung der Vereinten Nationen [6].

Der Sonderbericht widmet sich aber nicht nur den Konsequenzen fortschreitender Erwärmung. Er zeigt auch auf, welche Emissionspfade mit dem 1,5-°C- oder 2-°C-Ziel vereinbar sind. Hierbei zeigt sich, dass alle Pfade, welche die globale Erwärmung ohne oder mit geringer Überschreitung auf 1,5 °C begrenzen, schnelle und weitreichende Systemübergänge in vielen Bereichen der Wirtschaft und Gesellschaft erfordern. Weiters zeigen all diese Pfade auch die Notwendigkeit der Nutzung von Methoden der Kohlendioxidentnahme (engl. carbon dioxide removal, CDR). Hierbei nehmen die Netto-CO_2_-Emissionen bis 2030 um etwa 45 % gegenüber dem Niveau von 2010 ab und erreichen um das Jahr 2050 netto null. Szenario abhängig ist weiters CDR in der Größenordnung von 100–1000 Gt CO_2_ erforderlich, um verbleibende Emissionen auszugleichen bzw. die erforderlichen netto negativen Emissionen zu erzielen, um die globale Erwärmung nach einem Höchststand wieder auf 1,5 °C zu reduzieren [6].

Das Ausmaß der erforderlichen Emissionsreduktionen ist klar. Mit heutigem Stand sind jedoch deutlich weitreichendere Emissionsminderungen erforderlich, um das 1,5-°C-Ziel zu erreichen, als im Rahmen der nationalen Klimaaktionspläne eingereicht. Hierbei wird auch die Auswirkung jüngerer politischer Ereignisse, wie des Rückzugs der USA aus dem Pariser Abkommen oder der „Green New Deal“ der EU-Kommission Bedeutung erlangen. Die deutliche Mehrheit der Emissionspfade, welche die globale Erwärmung ohne oder mit geringer Überschreitung auf 1,5 °C begrenzen, zeigen bis 2030 einen Rückgang der globalen Treibhausgasemissionen auf weniger als 35 Gt CO_2_-Äquivalent pro Jahr [6]. Entscheidend hierfür ist die rasche Umsetzung und Ausweitung der Minderungsmaßnahmen, denn je geringer die Emissionen bei 2030 ausfallen, umso leichter ist die Begrenzung der globalen Erwärmung auf 1,5 °C.

## Aktuelle Entwicklungen

Wie vorangehend beschrieben, sind deutlich umfassendere Emissionsminderungen als bis dato im Rahmen der nationalen Klimaaktionspläne verankert erforderlich, um das 1,5-°C-Grad-Ziel zu erreichen. Erschwerend kommt hinzu, dass viele Nationen in ihren Emissionsreduktionen ihren Plänen hinterher hinken (https://climateactiontracker.org/, zuletzt zugegriffen am 17.0.9.2020). So war es bis vor kurzem auch nicht klar, ob z.B. Österreich seine Klimaziele für 2020 im Rahmen der EU Lastenteilung (engl. Effort Sharing) erreichen wird. Insgesamt ist entscheidend zu beachten, dass die Entwicklungspfade des Sonderberichts des IPCC zum 1,5-°C-Grad-Ziel netto null Emission um 2050 vorsehen. Um dieses Ziel zu erreichen, bleiben also gerade noch drei Jahrzehnte Zeit, jedoch auch nur dann, wenn die Emissionsminderung rasch eingeleitet wird und somit die höchstzulässigen Emissionsmengen nicht überschritten werden. Die Welt-CO_2_-Uhr (https://www.mcc-berlin.net/fileadmin/data/clock/carbon_clock.htm, zuletzt zugegriffen am 17.0.9.2020) steht auf kurz vor Zwölf, und läuft bei gleichbleibender globaler CO_2_-Emission von rund 1300 Tonnen pro Sekunde, noch in diesem Jahrzehnt für das 1,5-°C-Ziel ab.

Es scheint, dass mit Auftreten der COVID-19-Pandemie die Klimakrise an Aufmerksamkeit verloren hat. Aber auch mitten in der COVID-19-Pandemie werden uns die Folgen der fortschreitenden Erderhitzung durch eine Reihe von Extremereignissen deutlich vor Augen geführt. Der Klimawandel legt keine Pause ein.

Die COVID-19-Pandemie zeigt uns aber auch auf, wie umfassend sich unser Emissionsverhalten ändern muss. Durch den globalen Shutdown wurde das Verkehrsaufkommen und die industrielle Produktion stark reduziert. Als Folge wurden im April 2020 auf globaler Skala CO_2_-Emissionsabnahmen von ca. 17 % im Vergleich zum Vorjahr beobachtet [19]. Eine kürzlich erschienene Studie hat die Klimawirkung der Reduktion in Treibhausgasen und anderen Luftschadstoffen während des COVID-19 Shutdowns analysiert. Das Ergebnis scheint vielleicht auf den ersten Blick überraschend: Der direkte klimatische Effekt eines kurzfristigen COVID-19-Shutdowns mit nachfolgender Rückkehr zum vorherigen Emissionsniveau ist vernachlässigbar gering; bei 2030 zeigt sich hierbei lediglich eine Abkühlung von $0.01 \pm 0.005$ °C im Gegensatz zu Berechnungen ohne jeglichen Shutdown [20]. Die Ursache hierfür ist, dass kurzfristige Schwankungen im Emissionsverhalten bedingt durch die relativ lange Lebensdauer von Treibhausgasen zu keiner Abnahme, sondern lediglich einer geringeren Zuwachsrate der atmosphärischen Konzentration führen. Eindrücklich ersichtlich wird dies auch darin, dass im Mai diesen Jahres, also kurz nach dem Peak des globalen Shutdowns, am Mauna Loa Observatorium der National Oceanic and Atmospheric Administration mit einem Messwert von 417,1 ppm ein neuer Rekord der monatlichen CO_2_-Konzentration gemessen wurde. Die Erfahrung des COVID-19-Shutdowns gibt aber auch Hoffnung. So zeigen Simulationen, die keine Rückkehr zum alten Wirtschaftssystem inklusive seiner Treibhausgasemissionen, sondern einen Wandel als Folge ambitionierter „Green-Stimulus-Investitionen“ berücksichtigen, dass 0,3 °C an Erderwärmung vermieden und so das 1,5-°C-Ziel bis 2050 erreicht werden kann [20].

## Schlussfolgerungen

Die Bewältigung der Klimakrise ist die größte Herausforderung unserer Zeit. Der Klimawandel wirkt in alle Lebensbereiche, und der Grad, auf dem es uns gelingt, die Erderhitzung zu begrenzen, wird entscheidend die Lebenswirtlichkeit für Generationen bestimmen. Eine Vielzahl von wissenschaftlichen Studien hat sich mit der Rolle des Menschen im Klimasystem beschäftigt. Der Weltklimarat stützt sich in seinen Sachstandsberichten auf diese Fachliteratur, und sein Befund ist eindeutig: „Der Einfluss des Menschen auf das Klimasystem ist klar. Das ist offensichtlich aufgrund der ansteigenden Treibhausgaskonzentrationen in der Atmosphäre, dem positiven Strahlungsantrieb, der beobachteten Erwärmung und des Verständnisses des Klimasystems“ [4].

Um besonders schwerwiegende Klimafolgen zu vermeiden, wurde im Rahmen des Pariser Abkommens das Ziel ausgerufen, die Erderhitzung auf deutlich unter 2 °C, idealerweise auf 1,5 °C zu begrenzen. Um das 1,5 °C Ziel zu erreichen, sind rasche und umfassende Emissionsminderungen erforderlich, welche deutlich über die bis dato vereinbarten nationalen Klimaaktionspläne hinausgehen. Erforderlich sind Emissionsreduktionen bei 2030 von ca. minus 45 % gegenüber dem Niveau von 2010 und netto null Emissionen um 2050 [6]. Nüchtern betrachtet angesichts der historischen Entwicklung der Emissionen eine Mammutaufgabe, jedoch unvermeidlich, um eine nachhaltige Sicherung der Lebenswirtlichkeit auf unserem Planeten zu gewährleisten. Fest steht, dass zur Erreichung dieses Zieles umfassende Änderungen unserer technischen, ökonomischen und sozialen Systeme erforderlich sind, als zentrales Element Dekarbonisierung.

Autoren anderer Beiträge dieses Bandes befassen sich eingehend mit der Frage, welchen Beitrag Digitalisierung zur Erreichung der Klimaziele leisten kann. Die fortschreitende Digitalisierung bietet im Konnex zum Klimaschutz gleichzeitig Chancen (u.a. Emissionsminderung durch Veränderung von Prozessen in energieintensiven Branchen, Reduktion von Verkehrsemission) und Risiken (Emissionszuwachs durch Erzeugung zusätzlich benötigter elektrischer Energie aus fossilen Energieträgern). So sind zur nachhaltigen Umsetzung bei Digitalisierungsvorhaben Fragen der Klima- und Ressourcenschonung bereits in der Planung dringend zu berücksichtigen, Emissionsminderung zentral zu verankern, und sicherzustellen, dass keine negativen Lock-in-Effekte entstehen. Deutliche Emissionsminderung bei 2030 und Klimaneutralität bei 2050 ist erforderlich, um das 1,5-°C-Ziel zu erreichen. Noch ist die Begrenzung der Erderhitzung unter dieser Schwelle möglich. Maßnahmenpakete wie der „Green New Deal“ der EU-Kommission und ambitionierte Klimaziele einzelner Nationalstaaten geben Hoffnung. Entscheidend sind jedoch nicht die Ziele, sondern deren Erreichung. Eine effiziente und rasche Umsetzung von Klimaschutzmaßnahmen heute, nicht erst morgen, ist unerlässlich für eine gedeihliche Entwicklung unseres Planeten im Anthropozän.

## Footnotes

1 United Nations Framework Convention on Climate Change, 21st Conference of the Parties.

## References

[1] Crutzen, P. J. (2002): Geology of mankind. Nature, 415(6867), 23. 10.1038/415023a. 11780095 10.1038/415023a

[2] IPCC (2001): Zusammenfassung für politische Entscheidungsträger. In Klimaänderung 2001: Wissenschaftliche Grundlagen. Beitrag der Arbeitsgruppe I zum Dritten Sachstandsbericht des Zwischenstaatlichen Ausschusses für Klimaänderung (IPCC), Cambridge: Cambridge University Press. Deutsche Übersetzung online verfügbar unter https://www.ipcc.ch/site/assets/uploads/2019/03/2001-wg1_ge.pdf, zuletzt zugegriffen am 17.09.2020.

[3] IPCC (2007): IPCC 2007: Zusammenfassung für politische Entscheidungsträger. In Klimaänderung 2007: Wissenschaftliche Grundlagen. Beitrag der Arbeitsgruppe I zum Vierten Sachstandsbericht des Zwischenstaatlichen Ausschusses für Klimaänderung (IPCC). Cambridge: Cambridge University Press. Deutsche Übersetzung durch ProClim-, österreichisches Umwelt-bundesamt, deutsche IPCC-Koordinationsstelle, Bern/Wien/Berlin, 2007, online verfügbar unter https://naturwissenschaften.ch/service/publications/76776-ipcc-klimaaenderung-2007-zusammenfassungen-fuer-politische-entscheidungstraeger, zuletzt zugegriffen am 17.09.2020.

[4] IPCC (2013): Zusammenfassung für politische Entscheidungsträger. In Klimaänderung 2013: Naturwissenschaftliche Grundlagen. Beitrag der Arbeitsgruppe I zum Fünften Sachstandsbericht des Zwischenstaatlichen Ausschusses für Klimaänderungen (IPCC). Cambridge: Cambridge University Press. Deutsche Übersetzung durch Deutsche IPCC-Koordinierungsstelle, Österreichisches Umweltbundesamt, ProClim, Bonn/Wien/Bern, 2014, online verfügbar unter https://www.ipcc.ch/site/assets/uploads/2018/03/ar5-wg1-spmgerman.pdf, zuletzt zugegriffen am 17.09.2020.

[5] Cook, J., Oreskes, N., Doran, P. T., Anderegg, W. R. L., Verheggen, B., Maibach, E. W., Carlton, J. S., Lewandowsky, S., Skuce, A. G., Green, S. A., Nuccitelli, D., Jacobs, P., Richardson, M., Winkler, B., Painting, R., Rice, K. (2016): Consensus on consensus: a synthesis of consensus estimates on human-caused global warming. Environ. Res. Lett., 11(4), 048002. 10.1088/1748-9326/11/4/048002.

[6] IPCC (2018): Summary for policymakers. In V. Masson-Delmotte, P. Zhai, H. O. Pörtner, D. Roberts, J. Skea, P. R. Shukla, A. Pirani, W. Moufouma-Okia, C. Péan, R. Pidcock, S. Connors, J. B. R. Matthews, Y. Chen, X. Zhou, M. I. Gomis, E. Lonnoy, T. Maycock, M. Tignor, T. Waterfield (Hrsg.) Global warming of 1.5 °C. an IPCC special report on the impacts of global warming of 1.5 °C above pre-industrial levels and related global greenhouse gas emission pathways, in the context of strengthening the global response to the threat of climate change, sustainable development, and efforts to eradicate poverty. In Press, Deutsche Übersetzung online verfügbar unter https://www.ipcc.ch/site/assets/uploads/2020/07/SR1.5-SPM_de_barrierefrei.pdf, zuletzt zugegriffen am 17.09.2020.

[7] Friedlingstein, P., Jones, M.W., O’Sullivan, M., Andrew, R. M., Hauck, J., Peters, G. P., Peters, W., Pongratz, J., Sitch, S., Le Quéré, C., Bakker, D. C. E., Canadell, J. G., Ciais, P., Jackson, R. B., Anthoni, P., Barbero, L., Bastos, A., Bastrikov, V., Becker, M., Bopp, L., Buitenhuis, E., Chandra, N., Chevallier, F., Chini, L. P., Currie, K. I., Feely, R. A., Gehlen, M., Gilfillan, D., Gkritzalis, T., Goll, D. S., Gruber, N., Gutekunst, S., Harris, I., Haverd, V., Houghton, R. A., Hurtt, G., Ilyina, T., Jain, A. K., Joetzjer, E., Kaplan, J. O., Kato, E., Klein Goldewijk, K., Korsbakken, J. I., Landschützer, P., Lauvset, S. K., Lefèvre, N., Lenton, A., Lienert, S., Lombardozzi, D., Marland, G., McGuire, P. C., Melton, J. R., Metzl, N., Munro, D. R., Nabel, J. E. M. S., Nakaoka, S. I., Neill, C., Omar, A. M., Ono, T., Peregon, A., Pierrot, D., Poulter, B., Rehder, G., Resplandy, L., Robertson, E., Rödenbeck, C., Séférian, R., Schwinger, J., Smith, N., Tans, P. P., Tian, H., Tilbrook, F. N., van der Werf, G. R., Wiltshire, A. J., Zaehle, S. (2019): Global carbon budget 2019. Earth Syst. Sci. Data, 11(4), 1783–1838. 10.5194/essd-11-1783-2019.

[8] Friedlingstein, et al. (2019): Regional, and national fossil-fuel CO_2_ emissions. In D. Gilfillan, G. Marland, T. Boden, R. Andres, available at: https://energy.appstate.edu/CDIAC, last access: 27 September 2019, 2019.

[9] Nehrbass-Ahles, C., Shin, J., Schmitt, J., Bereiter, B., Joos, F., Schilt, A., Schmidely, L., Silva, L., Teste, G., Grilli, R., Chappellaz, J., Hodell, D., Fischer, H., Stocker, T. F. (2020): Abrupt CO_2_ release to the atmosphere under glacial and early interglacial climate conditions. Science, 369(6506), 1000–1005. 10.1126/science.aay8178. 32820127 10.1126/science.aay8178

[10] Vincent, C., Fischer, A., Mayer, C., Bauder, A., Galos, S. P., Funk, M., Thibert, E., Six, D., Braun, L., Huss, M. (2017): Common climatic signal from glaciers in the European Alps over the last 50 years. Geophys. Res. Lett., 44(3), 1376–1383. 10.1002/2016gl072094.

[11] Fischer, A., Seiser, B., Waldhuber, M. S., Mitterer, C., Abermann, J. (2015): Tracing glacier changes in Austria from the Little Ice Age to the present using a lidar-based high-resolution glacier inventory in Austria. Cryosphere, 9(2), 753–766. 10.5194/tc-9-753-2015.

[12] Neukom, R., Steiger, N., Gomez-Navarro, J. J., Wang, J. H., Werner, J. P. (2019): No evidence for globally coherent warm and cold periods over the preindustrial Common Era. Nature, 571, (7766):550–+. 10.1038/s41586-019-1401-2. 31341300 10.1038/s41586-019-1401-2

[13] Oreskes, N., Conway, E. M. (2010): Merchants of doubt: how a handful of scientists obscured the truth on issues from tobacco smoke to global warming. New York: Bloomsbury Press.

[14] Hasselmann, K. (2010): The climate change game. Nat. Geosci., 3(8), 511–512. 10.1038/ngeo919.

[15] Chimani, B., Heinrich, G., Hofstätter, M., Kerschbaumer, M., Kienberger, S., Leuprecht, A., Lexer, A., Peßenteiner, S., Poetsch, M. S., Salzmann, M., Spiekermann, R., Switanek, M. H. T. (2016): ÖKS15 – Klimaszenarien für Österreich. Daten, Methoden und Klimaanalyse. Wien: Projektendbericht.

[16] Arguez, A., Hurley, S., Inamdar, A., Mahoney, L., Sanchez-Lugo, A., Yang, L. (2020): Should we expect each year in the next decade (2019–28) to be ranked among the top 10 warmest years globally? Bull. Am. Meteorol. Soc., 101(5), E655–E663. 10.1175/bams-d-19-0215.1.

[17] NOAA (2020): NOAA national centers for environmental information, climate at a glance. Global time series. Published September 2020, retrieved on August 15, 2020 from https://www.ncdc.noaa.gov/cag/.

[18] Lenton, T. M., Held, H., Kriegler, E., Hall, J. W., Lucht, W., Rahmstorf, S., Schellnhuber, H. J. (2008): Tipping elements in the Earth’s climate system. Proc. Natl. Acad. Sci. USA, 105(6), 1786–1793. 10.1073/pnas.0705414105. 18258748 10.1073/pnas.0705414105 PMC2538841

[19] Le Quéré, C., Jackson, R. B., Jones, M. W., Smith, A. J. P., Abernethy, S., Andrew, R. M., De-Gol, A. J., Willis, D. R., Shan, Y., Canadell, J. G., Friedlingstein, P., Creutzig, F., Peters, G. P. (2020): Temporary reduction in daily global CO2 emissions during the COVID-19 forced confinement. Nat. Clim. Change, 10(7), 647–653. 10.1038/s41558-020-0797-x.

[20] Forster, P. M., Forster, H. I., Evans, M. J., Gidden, M. J., Jones, C. D., Keller, C. A., Lamboll, R. D., Quéré, C. L., Rogelj, J., Rosen, D., Schleussner, C-F., Richardson, T. B., Smith, C. J., Turnock, S. T. (2020): Current and future global climate impacts resulting from COVID-19. Nat. Clim. Change. 10.1038/s41558-020-0883-0. 10.1038/s41558-020-0904-z PMC7427494 32845944

